# Correlation between clinical severity and different non-invasive measurements of carbon monoxide concentration: A population study

**DOI:** 10.1371/journal.pone.0174672

**Published:** 2017-03-28

**Authors:** Thomas Hullin, Jerome Aboab, Kristell Desseaux, Sylvie Chevret, Djillali Annane

**Affiliations:** 1 Service de Réanimation Polyvalente, Hôpital Raymond Poincaré, AP-HP, Université de Versailles Saint-Quentin-en-Yvelines, Garches, France; 2 Service de Biostatistique et Information Médicale, Hôpital Saint Louis, Paris, France; University of Missouri Health Care, UNITED STATES

## Abstract

**Objectives:**

Carbon monoxide (CO) poisoning is a major concern in industrialized countries. Each year, thousands of victims, resulting in approximately 100 fatalities, are encountered in France. The diagnosis of CO poisoning is challenging; while carboxyhemoglobin (COHb) may be useful, it is a weak indicator of the severity of CO poisoning. This weak indicator may be a result of the delay between poisoning occurrence and the blood assay. Two apparatuses, CO oximeters and exhaled CO analyzers, now permit COHb to be determined outside hospitals. Our hypothesis is that these instruments allow the early measurement of COHb concentrations, which are more correlated with the severity of poisoning, expressed using the poisoning severity score (PSS).

**Design:**

In an observational and retrospective cohort study, the distribution of COHb measurements obtained by CO oximetry or by exhaled CO analyzers was compared between groups of severity expressed using the PSS.

**Setting:**

Data were collected in the Paris area from January 2006 to December 2010 by the French Surveillance System of CO poisoning.

**Participants:**

All patients with CO poisoning reported to the French Surveillance System of CO poisoning.

**Results:**

There was a significant difference in the COHb values obtained by CO oximetry between groups stratified according to PSS (p<0.0001). A significant difference in the values of exhaled CO was also observed between PSS groups (p = 0.006), although the relationship was not linear.

**Conclusions:**

The COHb concentrations measured using CO oximetry, but not those measured using exhaled CO analyzers, were well correlated with the severity of CO poisoning.

## Introduction

Carbon monoxide (CO) poisoning is the most common cause of accidental poisoning in France with approximately 4000 cases per year reported by the French CO Poisoning Surveillance System [1]. Consequences may be severe and include transient neurological symptoms, coma, respiratory and cardiovascular failure, and death. The diagnosis of CO poisoning is based on unspecific clinical features, measurement of carboxyhemoglobin (COHb) in the blood, and discovery of CO in the atmosphere on site. However, COHb is not correlated with the severity of CO poisoning [2] or with the occurrence of delayed neurological sequel (DNS) [3]. The delay between the end of exposure and measurement and the application of oxygen therapy before measurement are often cited to explain the lack of correlation. In France, the first responders (firefighters or emergency medical personnel) are now often equipped with CO breath analyzers or CO oximeters. These two apparatuses can provide information about CO poisoning levels without the need for a blood sample and within a short time after CO exposure. The CO breath analyzer provides the CO concentration in exhaled air, which is well correlated with low COHb values (<40%) [4]; however, it tends to underestimate high values [5]. CO oximeters allow the non-invasive measurement of COHb with precision between 2% and 6% [6–8]. These new instruments can allow the early determination of COHb, which may be more correlated to severity. The objective of this study was to evaluate the correlation between measurements obtained using breath analyzers or via CO oximetry and clinical severity among patients with CO poisoning.

## Methods

This study is an observational and multicenter cohort study. Data were provided by the French CO Poisoning Surveillance System coordinated by the Institute of Health Surveillance (“Institut de Veille Sanitaire—InVS”) to which cases of CO poisoning are declared. Any suspected or confirmed intoxication is reported to the health authority (regional health agency or anti-poison centre by delegation). Variables were assessed by the physician treating the victims of CO poisoning in an emergency room or in any hospital department. And a public health doctor of the regional health agency or anti-poison center collected the data using a standardized medical form. They send these forms on a web application to constitute the epidemiological database exploited by the French Surveillance System of CO poisoning. The French data protection agency (“Commission Nationale de l'Informatique et des Libertées—CNIL”) approved this retrospective study, and the data were completely anonymous in accordance with French regulation (approval number: 1375107). All cases of CO poisoning in Paris and suburbs reported to the French CO Poisoning Surveillance System from January 2006 to December 2010 were included. CO poisoning cases that were fire-related were excluded because toxins other than CO (e.g., cyanide) could have been involved. The data collected included demographic data (sex and age), pregnancy status, smoking status, and initial clinical features. The initial clinical features included headache, asthenia, nausea/vomiting, vertigo, transient loss of consciousness, transient palsy, dyspnea, coma, pulmonary edema, chest pain, ventricular arrhythmia, myocardial infarction, seizure, circulatory failure, rhabdomyolysis, severe acidosis, brain stroke, and death. The severity of the poisoning was established using the modified Poisoning Severity Score (PSS) developed by the European Association of Poison Centres and Clinical Toxicologists [9]. The modified PSS introduces a sixth grade, differentiating transient loss of consciousness and transient palsy from coma and stroke; this allows each patient to be classified into one of 6 severity grades: asymptomatic, 0; minor, 1; moderate, 2; intermediate, 3; severe, 4; and fatal, 5 (Table 1). Data regarding the nature of the treatment (normobaric or hyperbaric oxygen therapy) were also collected. Finally, the value of the biomarker with CO was collected before or after initiation of oxygen therapy. The value by CO oximetry was expressed as a percentage of the total hemoglobin concentration. Exhaled CO was expressed as ppm, and the value expressed by the blood COHb assay was expressed as a percentage of the total hemoglobin concentration.

**Table 1:** Poisoning severity score (adapted from Persson et al. [9]).

Grade	Sign
0	None
1	Asthenia, headache
2	Nausea / vomiting, vertigo
3	Transient loss of consciousness, transient palsy, dyspnea
4	Pulmonary edema, chest pain, ventricular arrhythmia, myocardial infarction, seizure, circulatory failure, rhabdomyolysis, severe acidosis, brain stroke
5	Death

The data provided by the InVS were anonymous; thus, it was impossible to check or obtain other information from the medical record of patients.

Quantitative results were expressed as medians with interquartile ranges and qualitative results as percentages. Univariate comparisons were made using the exact Fisher test (for qualitative or discrete variables) or the Wilcoxon rank sum test (for continuous variables). Then, the distribution of CO impregnation was compared between groups of increasing clinical severity, i.e., severity grade, by a non-parametric Kruskal-Wallis test. To control for the potential confounding effect of smoking, we further adjusted these comparisons on smoking status, using a linear model. All p-values were two-sided, with p<0.05 denoting statistical significance. The statistical analysis was performed using R^®^ v2.15.2 (http://www.R-project.org/).

## Results

The study included 3153 patients. Table 2 shows the patient and CO measurement characteristics. The median age was 31 (interquartile range, 13–44) years. Fifty one percent of patients were female. Smoking status was available in 2060 patients with 1685 (53%) being non-smokers and 375 (12%) being smokers. A total of 125 (3.9%) patients were lost to follow-up after intoxication. Among the 3028 remaining patients, 31 (0.98%) died, with death occurring at the place of the intoxication in 15 patients (including 3 after the first responders arrived), in the hospital in 6 cases, and at unknown locations in the remaining 10 cases. Of those treated with oxygen, 2196 patients received treatment with normobaric oxygen alone, and 408 patients received treatment with hyperbaric oxygen. The remaining 443 patients received no oxygen therapy.

**Table 2:** Patient and CO measurement characteristics.

Characteristic		Number of patient	% or median [IQR]
**Population**		3153	
**Age (years)**		2948	31 [13–44]
**Gender**	male	1501	49%
	female	1560	51%
	unknown	92	
**Smoking status**	no	1685	53%
	yes	375	12%
	unknown	1093	35%
**Clinical severity**	0	1543	49%
	1	848	27%
	2	485	15%
	3	209	7%
	4	37	1%
	5	31	1%
**Exhaled CO measurement**		94	3%
**Exhaled CO (ppm)**	all	94	51.5 [10–137]
	before oxygen therapy	64	72.50 [12.75–144.8]
	after oxygen therapy	4	43 [7.5–124.5]
	unknown	26	22.50 [6.25–113.8]
**CO oximetry measurement**		90	3%
**CO oximetry (%COHb)**	all	90	15.85 [8–24.3]
	before oxygen therapy	23	16 [11–26]
	after oxygen therapy	58	15.35 [8–22]
	unknown	9	16 [12–26]
**Blood assay measurement**		2328	74%
**Blood assay (%COHb)**	all	2327*	8 [3.6–14.3]
	before oxygen therapy	1382	8.9 [4.1–15.2]
	after oxygen therapy	495	7.5 [3.5–13.9]
	unknown	451	6.4 [2.2–11]
**Evolution**	alive	2997	95%
	deceased	31	1%
	unknown	125	4%
**Place of death**	out of hospital before first responder arrival	12	57%
	out of hospital after first responder arrival	3	14%
	in hospital	6	29%
**Hospitalization**	no	2470	79%
	yes	606	19%
	unknown	77	2%

Data are median [Q1; Q3] or number of patients (%);

*: one patient had a blood assay performed but the value was missing.

The PSS was 0, 1, 2, 3, 4, and 5 for 1543 (48.9%), 848 (26.9%), 485 (15.4%), 209 (6.6%), 37 (1.2%), and 31 (1%) patients, respectively.

**Fig 1:**
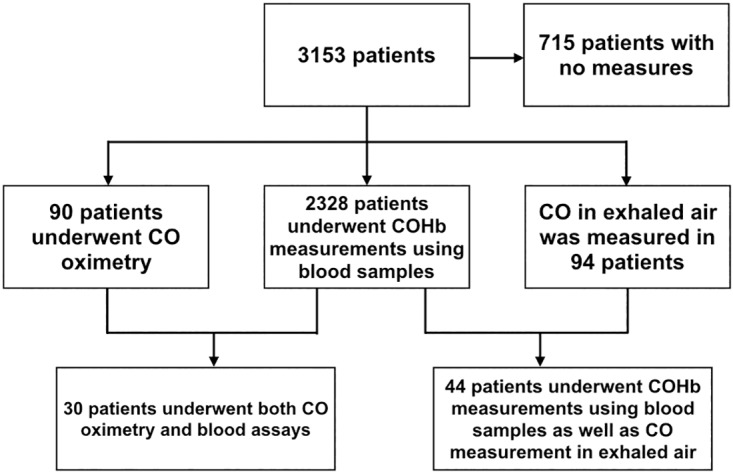
Flow chart showing the types of assays performed.

Among the 3153 patients included, COHb was measured using blood samples in 2328 (73.8%) cases, CO was measured in exhaled breath 94 (3%) cases, and COHb was measured by CO oximetry in 90 (2.9%) cases. Some patients underwent two types of assessment. There were 715 (22.7%) patients who did not undergo any assessment (Fig 1).

**Fig 2:**
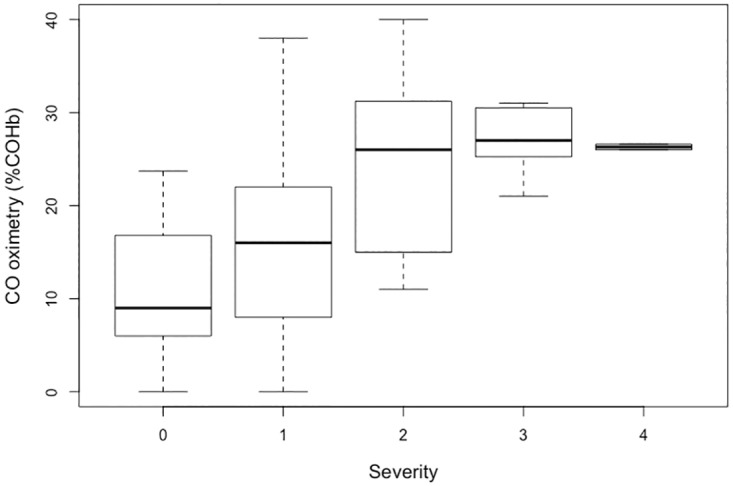
Relationship between COHb measured by CO oximetry (in %) and PSS.

Patients whose COHb concentrations were measured by CO oximetry were significantly more often smokers (18.9% vs 11.7%) and lost consciousness more frequently (12.2% vs 6.4%) than patients whose COHb concentrations were not measured by CO oximetry. There was a difference in the results of COHb by CO oximetry between the PSS groups (p<0.0001 by Kruskal-Wallis test) (Fig 2). This difference remained significant after adjustment for smoking status.

**Fig 3:**
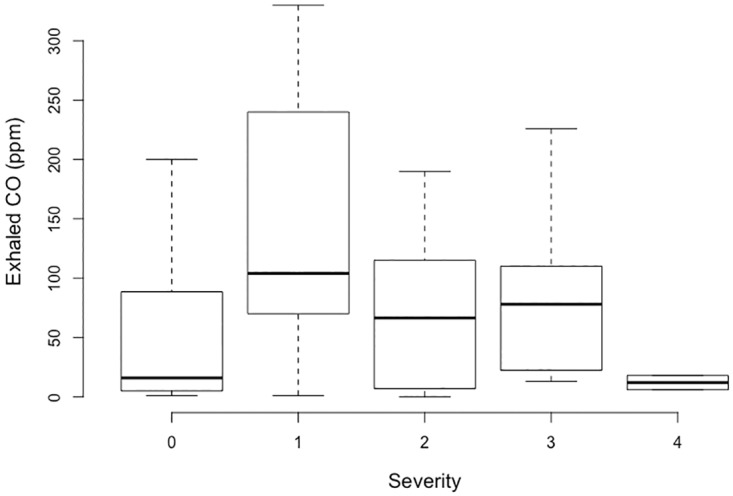
Relationship between exhaled CO (in ppm) and PSS.

Patients in whom CO in exhaled breath was assessed were significantly older (median age, 36 vs 31 years) and less often smokers (4.3% vs 12.1%) compared to those in whom CO in exhaled breath was not assessed. There was a significant difference in the results of exhaled CO between PSS groups (p = 0.006 by Kruskal-Wallis test), although the observed relationship appeared to be not linear (Fig 3).

**Fig 4:**
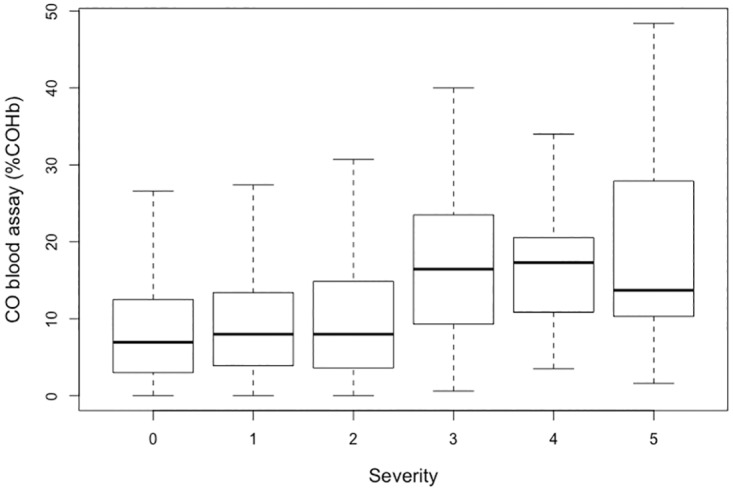
Relationship between COHb measurement by blood assay (in %) and PSS.

Patients whose blood samples were used for COHb assessment were significantly more often smokers (13.4% vs 7.5%) and more frequently lost consciousness (7% vs 5.2%) than patients whose blood samples were not used for COHb measurement. There was a significant difference in the values of COHb between PSS groups (p<0.0001 by Kruskal-Wallis test) (Fig 4). The difference remained significant after adjustment for smoking status.

## Discussion

To our knowledge, no previous clinical study found a strong correlation between CO values measured by CO oximetry and the clinical severity of CO poisoning. This is the first study to identify such a correlation. CO oximetry provides an objective indication of the clinical severity of poisoning. Therefore, during the initial clinical assessment, CO oximetry could help to identify the most severe poisoning cases. In cases of mixed intoxication such as those noted in cases of suicide attempts with inhalation of exhaust gases and drug absorption, CO oximetry could help to distinguish the different toxins involved.

No study has evaluated the accuracy of measuring COHb with CO oximetry in cases of circulatory failure. In our study, the COHb values obtained for severity group 3 by oximetry were higher than those obtained for group 4 (28.25% and 26.3%, respectively). Although this difference is not significant, it is possible that the hemodynamic failure induced an underestimation of COHb by CO oximetry, as already reported for oxyhemoglobin by standard pulse oximetry [10].

No significant correlation was found in this study between exhaled CO concentration and clinical severity before clinical oxygen therapy regardless of the smoking status. The mean concentration of exhaled CO was even lower in groups 3 and 4 than that in groups with lower severity. Lapostolle et al. found a correlation between exhaled CO values and clinical severity [11]. The main hypothesis to explain these conflicting results is that the procedure needed to obtain a good measurement of exhaled CO was not controlled in this study, whereas it was controlled in the study by Lapostolle et al. Although the methodology of Lapostolle's study implied that the measurement was performed appropriately, this measurement requires the achievement of apnea for 20 seconds and full expiration into the manifold. Patients with CO poisoning suffering from neurological impairment cannot comply with complex techniques of breath analysis. Therefore, this technique will be inappropriate in such patients. In contrast, CO oximetry does not rely on the patient's cooperation and therefore can be used on CO poisoning patients with neurological impairment and non-cooperative patients such as children.

A few limitations should be highlighted. The delay between the end of exposure to CO and the measurement of CO levels (by breath analyzer or CO oximetry) was unknown. This is explained by the limited data provided to us by the InVS. Consequently, the levels may have been underestimated compared to the initial level. However, the patients included in this study lived in an urban area. Hence, the delay should have been short. As the half-life of COHb is approximately 4 hours when breathing air, we suppose that measurements obtained before oxygen therapy were sufficiently accurate. Despite the large number of patients included, fewer than 100 measurements were available for each technique. The reason may be the poor availability of those techniques or missing data in the CO poisoning case reports. Another explanation is that those techniques are used more often onsite by emergency medical teams than in emergency departments where standard oximetry using blood samples is available. Usually, patients with CO poisoning do not need an emergency medical team onsite and are transported to an emergency department by first responders (mainly firefighters in France).

The first study on the correlation between the COHb concentration and symptoms of CO poisoning dates back to the late 19^th^ century. Haldane [12], Sayers [13], and Killick [14] performed experimental studies on a small number of healthy volunteers (1 healthy volunteer for Killick, 3 for Sayers, and Haldane practiced the study on himself). These authors concluded that there was a good correlation between symptoms and COHb concentration. However, clinical studies assessing correlations between COHb concentration and the clinical severity of CO poisoning have used varying methods with contrasting results. Roche et al. found that COHb concentrations higher than 50 ml/L were associated with poorer prognosis than lower values [15]. Norkool [16], Blettery [17], Mathieu [18], and Meulemans [19] found a significant difference in carboxyhemoglobin level between patients who did or did not lose consciousness. However, Sokal [20], Burney [21], and Fang [22] did not find any significant correlation between clinical severity and COHb concentration. In most of these studies, the time elapsed between CO exposure and performance of the COHb blood assay was unknown, as was the duration between treatment initiation and the time of measurement. The time interval between exposure and measurement is probably the main explanation for the lack of correlation found in the literature with respect to our study.

## Conclusion

In cases of CO poisoning, COHb concentrations measured by CO oximetry strongly correlated with clinical severity. No significant correlation was found between values of exhaled CO and clinical severity, probably because of the more restrictive measurement technique especially among patients unable to comply with breath analyzers procedure. CO oximetry should be prioritized over the use of breath analyzers for diagnosing CO poisoning in emergency departments and first responder units.

## Supporting information

S1 Dataset Data study. Anonymized data from the study, case number and year of poisoning have been erased for confidentiality purpose.
(XLS)

## References

[1] Verrier A, Delaunay C, Coquet S. Les intoxications au monoxyde de carbone survenues en France métropolitaine en 2007. Bull Épidémiologique Hebd. 2010;(1):1.

[2] Hampson NB, Hauff NM. Carboxyhemoglobin levels in carbon monoxide poisoning: do they correlate with the clinical picture? Am J Emerg Med. 2008;26(6):665–9. 10.1016/j.ajem.2007.10.005 18606318

[3] Raphael JC, Elkharrat D, Jars-Guincestre MC, Chastang C, Chasles V, Vercken JB, et al Trial of normobaric and hyperbaric oxygen for acute carbon monoxide intoxication. Lancet. 19 août 1989;2(8660):414–9. 2569600 10.1016/s0140-6736(89)90592-8

[4] Wald NJ, Idle M, Boreham J, Bailey A. Carbon monoxide in breath in relation to smoking and carboxyhaemoglobin levels. Thorax. 1981;36(5):366–9. 7314006 10.1136/thx.36.5.366 PMC471511

[5] Cunnington AJ, Hormbrey P. Breath analysis to detect recent exposure to carbon monoxide. Postgrad Med J. 2002;78(918):233–7. 10.1136/pmj.78.918.233 11930027 PMC1742317

[6] Barker SJ, Curry J, Redford D, Morgan S. Measurement of carboxyhemoglobin and methemoglobin by pulse oximetry: a human volunteer study. Anesthesiology. 2006;105(5):892–7. 17065881 10.1097/00000542-200611000-00008

[7] Piatkowski A, Ulrich D, Grieb G, Pallua N. A new tool for the early diagnosis of carbon monoxide intoxication. Inhal Toxicol. 2009;21(13):1144–7. 10.3109/08958370902839754 19852557

[8] Touger M, Birnbaum A, Wang J, Chou K, Pearson D, Bijur P. Performance of the RAD-57 pulse CO-oximeter compared with standard laboratory carboxyhemoglobin measurement. Ann Emerg Med. 2010;56(4):382–8. 10.1016/j.annemergmed.2010.03.041 20605259

[9] Persson HE, Sjöberg GK, Haines JA, Pronczuk de Garbino J. Poisoning severity score. Grading of acute poisoning. J Toxicol Clin Toxicol. 1998;36(3):205–13. 9656975 10.3109/15563659809028940

[10] Van de Louw A, Cracco C, Cerf C, Harf A, Duvaldestin P, Lemaire F, et al Accuracy of pulse oximetry in the intensive care unit. Intensive Care Med. 2001;27(10):1606–13. 10.1007/s001340101064 11685301

[11] Lapostolle F, Raynaud PJ, Le Toumelin P, Benaissa A, Agostinucci JM, Adnet F, et al [Measurement of carbon monoxide in expired breath in prehospital management of carbon monoxide intoxication]. Ann Fr Anesthèsie Rèanimation. 2001;20(1):10–5. 10.1016/s0750-7658(00)00340-3 11234571

[12] Haldane J. The action of carbonic oxide on man. J Physiol. 1895;18(5–6):430–62. 16992272 10.1113/jphysiol.1895.sp000578 PMC1514663

[13] Sayers R, Meriwether F, Yant W. Physiological effects of expoure to low concentration of carbon monxide. Public Health. 1922;37:1127–42.

[14] Killick EM. The acclimatization of the human subject to atmospheres containing low concentrations of carbon monoxide. J Physiol. 1936;87(1):41–55. 16994775 10.1113/jphysiol.1936.sp003387 PMC1395092

[15] Roche L, Bertoye A, Vincent P, Motin J, Garin JP, Bolot JF, et al [Comparison of 2 groups of 20 cases of carbon monoxide poisoning treated with normobaric and hyperbaric oxygen]. Lyon Méd. 1968;220(49):1483–99. 5714518

[16] Norkool DM, Kirkpatrick JN. Treatment of acute carbon monoxide poisoning with hyperbaric oxygen: a review of 115 cases. Ann Emerg Med. 1985;14(12):1168–71. 4061988 10.1016/s0196-0644(85)81023-4

[17] Blettery B, Virot C, Janoray P, Piganiol G. [Acute carbon monoxide poisoning in the emergency service. The importance of early signs. 90 cases]. Ann Médecine Interne. 1983;134(2):99–101. 6881806

[18] Mathieu D, Nolf M, Durocher A, Saulnier F, Frimat P, Furon D, et al Acute carbon monoxide poisoning. Risk of late sequelae and treatment by hyperbaric oxygen. J Toxicol Clin Toxicol. 1985;23(4–6):315–24. 4057322 10.3109/15563658508990639

[19] Meulemans A, Vanderwegen L, Sabbe M. Carbon monoxide and the emergency physician. J Eur Urg. 1993;6:124–33.

[20] Sokal JA, Kralkowska E. The relationship between exposure duration, carboxyhemoglobin, blood glucose, pyruvate and lactate and the severity of intoxication in 39 cases of acute carbon monoxide poisoning in man. Arch Toxicol. 1985;57(3):196–9. 4062554 10.1007/BF00290887

[21] Burney RE, Wu SC, Nemiroff MJ. Mass carbon monoxide poisoning: clinical effects and results of treatment in 184 victims. Ann Emerg Med. 1982;11(8):394–9. 7103154 10.1016/s0196-0644(82)80033-4

[22] Fang G, Xu G, Wang F, Hua B. Clinical significance of monitoring blood carboxyhemoglobin. J Hyperbaric Med. 1986;1(4):233–8.

